# 1-(2-Naphth­yl)-3-phenyl­prop-2-en-1-one

**DOI:** 10.1107/S1600536809025483

**Published:** 2009-07-04

**Authors:** Si-Ping Tang, Dai-Zhi Kuang, Yong-Lan Feng, Man-Sheng Chen, Wei Li

**Affiliations:** a Key Laboratory of Functional Organometallic Materials, Hengyang Normal University, Hengyang, Hunan 421008, People’s Republic of China

## Abstract

The title compound, C_19_H_14_O, contains two independent mol­ecules with the same *s-cis* conformation for the ketone unit. Both mol­ecules are non-planar with dihedral angles of 51.9 (1) and 48.0 (1)° between the benzene ring and the naphthalene ring system. In the crystal, neighboring mol­ecules are stabilized by intermolecular C—H⋯π inter­actions, giving a two-dimensional supra­molecular array parallel to the *ab* plane.

## Related literature

For background to chalcone and its derivatives, see: Agrinskaya *et al.* (1999 ▶); Indira *et al.* (2002 ▶); Opletalova (2000 ▶); Pandey *et al.* (2005 ▶). For related structures, see: Moorthi *et al.* (2005 ▶); Tang *et al.* (2008 ▶).
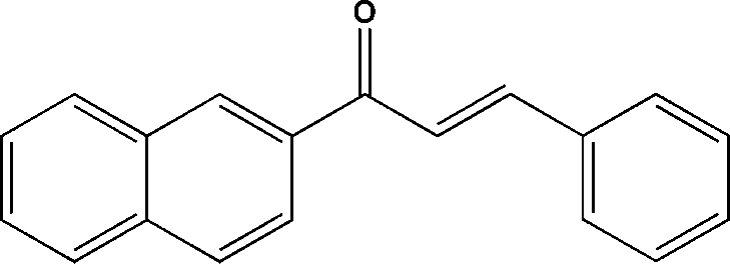


## Experimental

### 

#### Crystal data

C_19_H_14_O
*M* *_r_* = 258.30
Triclinic, 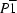

*a* = 9.5878 (8) Å
*b* = 9.6111 (8) Å
*c* = 15.5358 (13) Å
α = 98.746 (2)°
β = 91.222 (2)°
γ = 105.764 (1)°
*V* = 1358.8 (2) Å^3^
*Z* = 4
Mo *K*α radiation
μ = 0.08 mm^−1^
*T* = 295 K
0.30 × 0.28 × 0.20 mm

#### Data collection

Bruker SMART APEX area-detector diffractometer
Absorption correction: multi-scan (*SADABS*; Sheldrick, 1996 ▶) *T*_min_ = 0.972, *T*_max_ = 0.982
10623 measured reflections
5271 independent reflections
3483 reflections with *I* > 2σ(*I*)
*R*_int_ = 0.017

#### Refinement

*R*[*F* ^2^ > 2σ(*F* ^2^)] = 0.056
*wR*(*F* ^2^) = 0.174
*S* = 1.03
5271 reflections
361 parameters
H-atom parameters constrained
Δρ_max_ = 0.24 e Å^−3^
Δρ_min_ = −0.19 e Å^−3^

### 

Data collection: *SMART* (Bruker, 2002 ▶); cell refinement: *SAINT* (Bruker, 2002 ▶); data reduction: *SAINT*; program(s) used to solve structure: *SHELXS97* (Sheldrick, 2008 ▶); program(s) used to refine structure: *SHELXL97* (Sheldrick, 2008 ▶); molecular graphics: *SHELXTL* (Sheldrick, 2008 ▶); software used to prepare material for publication: *SHELXTL*.

## Supplementary Material

Crystal structure: contains datablocks I, global. DOI: 10.1107/S1600536809025483/at2828sup1.cif

Structure factors: contains datablocks I. DOI: 10.1107/S1600536809025483/at2828Isup2.hkl

Additional supplementary materials:  crystallographic information; 3D view; checkCIF report

## Figures and Tables

**Table 1:** Hydrogen-bond geometry (Å, °) *Cg*1, *Cg*2, *Cg*3, *Cg*5 and *Cg*6 are the centroids of the C1–C10, C5–C10, C14–C19, C20–C29 and C24–C29 benzene rings, respectively.

*D*—H⋯*A*	*D*—H	H⋯*A*	*D*⋯*A*	*D*—H⋯*A*
C13—H13⋯O1	0.93	2.49	2.814 (2)	101
C32—H32⋯O2	0.93	2.50	2.820 (2)	101
C18—H18⋯*Cg*1^i^	0.93	2.98	3.644	130
C15—H15⋯*Cg*2^ii^	0.93	2.94	3.642	134
C37—H37⋯*Cg*3^iii^	0.93	2.96	3.610	128
C1—H1⋯*Cg*5^ii^	0.93	2.97	3.611	127
C6—H6⋯*Cg*6^iii^	0.93	2.92	3.583	130

Symmetry codes: (i) 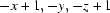; (ii) 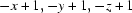; (iii) 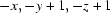.

**Fig. 1.**
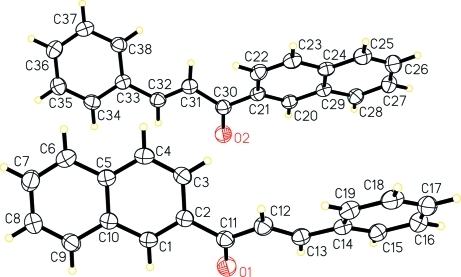
The title molecule with displacement ellipsoids drawn at the 30% probability level, and H atoms as spheres of arbitrary radius.

**Fig. 2.**
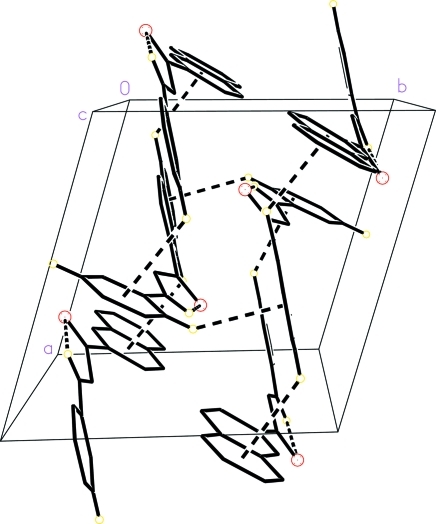
Packing diagram of the title structure showing the C—H···O and C—H···π interactions. The H atoms not involved in hydrogen bonding have been omitted for clarity.

## supplementary crystallographic information

### Comment

Chalcone and its analogues have been extensively researched because of their facile synthesis and potential applications as excellent non-linear optical materials (Agrinskaya *et al.*, 1999; Indira *et al.*, 2002) and biological activities (Opletalova, 2000; Pandey *et al.*, 2005). Ongoing our efforts on the research of chalcone compounds (Tang *et al.*, 2008), a new compound was here presented.

As shown in Fig.1, the title molecule contains two independent and isostructural molecules, which are non-planar because of the serious tilts between the benzene and naphthalene rings of 51.9 (1) ° and 48.0 (1) ° dihedral angles, respectively. In the two molecules, the ketone units display the same *s-cis.* conformations with the torsion angles of 14.7 (3) ° and 12.3 (3) °, respectively. Meanwhile, the intramolecular C—H···O hydrogen bonds exist within the ketone units, which are also found in the other similar structures (Moorthi *et al.*, 2005; Tang *et al.*, 2008).

In the crystal structure, as shown in Fig.2, neighboring molecules are stacked into a two-dimensional supramolecular layer by intermolecular C—H···π interactions parallel to the *ab* plane.

### Experimental

A mixture of 2-acetonaphthone (1.70 g, 10.0 mmol) and benzaldehyde (1.069 g, 10.0 mmol), sodium hydroxide (0.40 g, 10.0 mmol), ethanol (20 ml) and water (10 ml) was stirred at room temperature for 10 h. After filtering, the resulting precipitate was washed with water and iced ethanol, and further recrystallized from acetonitrile to afford colourless block crystals of the title compound [yield: 1.22 g (47.2 %)].

### Refinement

All H-atoms were positioned geometrically and refined using a riding model with d(C-H) = 0.93 Å, U_iso_=1.2U_eq_ (C).

### Crystal data

**Table:**

C_19_H_14_O	*Z* = 4
*M**_r_* = 258.30	*F*(000) = 544
Triclinic, *P*1	*D*_x_ = 1.263 Mg m^−^^3^
Hall symbol: -P 1	Mo *K*α radiation, λ = 0.71073 Å
*a* = 9.5878 (8) Å	Cell parameters from 2584 reflections
*b* = 9.6111 (8) Å	θ = 2.2–26.9°
*c* = 15.5358 (13) Å	µ = 0.08 mm^−^^1^
α = 98.746 (2)°	*T* = 295 K
β = 91.222 (2)°	Block, colourless
γ = 105.764 (1)°	0.30 × 0.28 × 0.20 mm
*V* = 1358.8 (2) Å^3^	

### Data collection

**Table:**

Bruker SMART APEX area-detector diffractometer	5271 independent reflections
Radiation source: fine-focus sealed tube	3483 reflections with *I* > 2σ(*I*)
graphite	*R*_int_ = 0.017
φ and ω scans	θ_max_ = 26.0°, θ_min_ = 2.2°
Absorption correction: multi-scan (*SADABS*; Sheldrick, 1996)	*h* = −11→11
*T*_min_ = 0.972, *T*_max_ = 0.982	*k* = −11→11
10623 measured reflections	*l* = −16→19

### Refinement

**Table:**

Refinement on *F*^2^	Primary atom site location: structure-invariant direct methods
Least-squares matrix: full	Secondary atom site location: difference Fourier map
*R*[*F*^2^ > 2σ(*F*^2^)] = 0.056	Hydrogen site location: inferred from neighbouring sites
*wR*(*F*^2^) = 0.174	H-atom parameters constrained
*S* = 1.03	*w* = 1/[σ^2^(*F*_o_^2^) + (0.0943*P*)^2^ + 0.07*P*] where *P* = (*F*_o_^2^ + 2*F*_c_^2^)/3
5271 reflections	(Δ/σ)_max_ < 0.001
361 parameters	Δρ_max_ = 0.24 e Å^−^^3^
0 restraints	Δρ_min_ = −0.19 e Å^−^^3^

### Special details

**Table:**

Geometry. All esds (except the esd in the dihedral angle between two l.s. planes) are estimated using the full covariance matrix. The cell esds are taken into account individually in the estimation of esds in distances, angles and torsion angles; correlations between esds in cell parameters are only used when they are defined by crystal symmetry. An approximate (isotropic) treatment of cell esds is used for estimating esds involving l.s. planes.
Refinement. Refinement of F^2^ against ALL reflections. The weighted R-factor wR and goodness of fit S are based on F^2^, conventional R-factors R are based on F, with F set to zero for negative F^2^. The threshold expression of F^2^ > 2sigma(F^2^) is used only for calculating R-factors(gt) etc. and is not relevant to the choice of reflections for refinement. R-factors based on F^2^ are statistically about twice as large as those based on F, and R- factors based on ALL data will be even larger.

### Fractional atomic coordinates and isotropic or equivalent isotropic displacement parameters (Å^2^)

**Table:**

	*x*	*y*	*z*	*U*_iso_*/*U*_eq_	
C1	0.55561 (16)	0.34700 (16)	0.30964 (10)	0.0513 (4)	
H1	0.6529	0.3967	0.3072	0.062*	
C2	0.50388 (16)	0.31927 (16)	0.38862 (10)	0.0512 (4)	
C3	0.35522 (17)	0.24451 (16)	0.39193 (11)	0.0564 (4)	
H3	0.3193	0.2228	0.4449	0.068*	
C4	0.26443 (17)	0.20422 (17)	0.31847 (11)	0.0569 (4)	
H4	0.1666	0.1581	0.3224	0.068*	
C5	0.31541 (16)	0.23088 (15)	0.23681 (10)	0.0492 (4)	
C6	0.22432 (18)	0.18909 (17)	0.15896 (12)	0.0617 (4)	
H6	0.1260	0.1428	0.1611	0.074*	
C7	0.2783 (2)	0.2156 (2)	0.08125 (12)	0.0730 (5)	
H7	0.2168	0.1879	0.0308	0.088*	
C8	0.4258 (2)	0.2841 (2)	0.07654 (12)	0.0735 (5)	
H8	0.4618	0.3014	0.0229	0.088*	
C9	0.51710 (19)	0.32576 (18)	0.14943 (11)	0.0634 (5)	
H9	0.6152	0.3705	0.1451	0.076*	
C10	0.46492 (16)	0.30194 (15)	0.23190 (10)	0.0493 (4)	
C11	0.60363 (17)	0.37413 (17)	0.46910 (11)	0.0556 (4)	
C12	0.57387 (18)	0.29557 (18)	0.54382 (11)	0.0598 (4)	
H12	0.5089	0.2024	0.5359	0.072*	
C13	0.63803 (17)	0.35515 (17)	0.62227 (11)	0.0548 (4)	
H13	0.6999	0.4496	0.6276	0.066*	
C14	0.62209 (15)	0.28857 (16)	0.70127 (11)	0.0510 (4)	
C15	0.67187 (17)	0.37583 (18)	0.78179 (11)	0.0581 (4)	
H15	0.7170	0.4753	0.7845	0.070*	
C16	0.65513 (19)	0.3166 (2)	0.85796 (12)	0.0693 (5)	
H16	0.6880	0.3765	0.9114	0.083*	
C17	0.59008 (19)	0.1694 (2)	0.85492 (13)	0.0728 (5)	
H17	0.5791	0.1297	0.9062	0.087*	
C18	0.54137 (18)	0.08120 (19)	0.77577 (13)	0.0678 (5)	
H18	0.4979	−0.0184	0.7737	0.081*	
C19	0.55654 (17)	0.13930 (17)	0.69964 (12)	0.0596 (4)	
H19	0.5228	0.0785	0.6466	0.071*	
C20	0.17274 (16)	0.87506 (16)	0.70822 (10)	0.0511 (4)	
H20	0.2253	0.9728	0.7122	0.061*	
C21	0.11862 (16)	0.79474 (16)	0.62779 (10)	0.0522 (4)	
C22	0.03921 (17)	0.64537 (17)	0.62222 (11)	0.0590 (4)	
H22	0.0000	0.5909	0.5682	0.071*	
C23	0.01976 (17)	0.58105 (17)	0.69474 (11)	0.0592 (4)	
H23	−0.0299	0.4822	0.6894	0.071*	
C24	0.07335 (16)	0.66125 (16)	0.77786 (11)	0.0509 (4)	
C25	0.05131 (18)	0.59894 (18)	0.85505 (12)	0.0639 (5)	
H25	0.0025	0.5001	0.8513	0.077*	
C26	0.10026 (19)	0.6810 (2)	0.93437 (12)	0.0701 (5)	
H26	0.0844	0.6380	0.9843	0.084*	
C27	0.17445 (19)	0.8298 (2)	0.94162 (12)	0.0675 (5)	
H27	0.2066	0.8853	0.9964	0.081*	
C28	0.19983 (17)	0.89384 (17)	0.86894 (11)	0.0584 (4)	
H28	0.2505	0.9925	0.8745	0.070*	
C29	0.15019 (15)	0.81224 (16)	0.78502 (10)	0.0489 (4)	
C30	0.14645 (18)	0.86629 (18)	0.54882 (11)	0.0586 (4)	
C31	0.04008 (18)	0.80888 (19)	0.47213 (11)	0.0641 (5)	
H31	−0.0495	0.7444	0.4786	0.077*	
C32	0.06982 (18)	0.84735 (17)	0.39480 (11)	0.0570 (4)	
H32	0.1619	0.9090	0.3907	0.068*	
C33	−0.02592 (17)	0.80371 (16)	0.31523 (10)	0.0519 (4)	
C34	0.03151 (18)	0.83379 (17)	0.23635 (11)	0.0590 (4)	
H34	0.1296	0.8816	0.2351	0.071*	
C35	−0.0556 (2)	0.79344 (19)	0.16030 (12)	0.0668 (5)	
H35	−0.0160	0.8138	0.1080	0.080*	
C36	−0.2010 (2)	0.72311 (18)	0.16094 (12)	0.0664 (5)	
H36	−0.2594	0.6956	0.1092	0.080*	
C37	−0.25980 (18)	0.69361 (17)	0.23827 (12)	0.0628 (5)	
H37	−0.3582	0.6465	0.2388	0.075*	
C38	−0.17429 (17)	0.73317 (17)	0.31472 (11)	0.0581 (4)	
H38	−0.2153	0.7129	0.3667	0.070*	
O1	0.70779 (13)	0.48236 (13)	0.47157 (8)	0.0757 (4)	
O2	0.25405 (14)	0.96832 (14)	0.54733 (8)	0.0820 (4)	

### Atomic displacement parameters (Å^2^)

**Table:**

	*U*^11^	*U*^22^	*U*^33^	*U*^12^	*U*^13^	*U*^23^
C1	0.0439 (8)	0.0479 (8)	0.0599 (10)	0.0099 (6)	0.0067 (7)	0.0072 (7)
C2	0.0504 (9)	0.0477 (8)	0.0545 (9)	0.0131 (7)	0.0059 (7)	0.0058 (7)
C3	0.0554 (9)	0.0568 (9)	0.0559 (10)	0.0126 (7)	0.0119 (8)	0.0105 (7)
C4	0.0468 (9)	0.0538 (9)	0.0677 (11)	0.0100 (7)	0.0082 (8)	0.0096 (8)
C5	0.0478 (8)	0.0433 (8)	0.0577 (10)	0.0160 (6)	0.0038 (7)	0.0060 (7)
C6	0.0529 (9)	0.0570 (9)	0.0723 (12)	0.0134 (7)	−0.0040 (8)	0.0059 (8)
C7	0.0762 (13)	0.0800 (12)	0.0596 (11)	0.0207 (10)	−0.0071 (9)	0.0047 (9)
C8	0.0792 (13)	0.0863 (13)	0.0551 (11)	0.0230 (10)	0.0082 (9)	0.0115 (9)
C9	0.0600 (10)	0.0676 (10)	0.0620 (11)	0.0160 (8)	0.0134 (8)	0.0114 (8)
C10	0.0498 (9)	0.0432 (8)	0.0561 (9)	0.0150 (6)	0.0063 (7)	0.0071 (7)
C11	0.0532 (9)	0.0548 (9)	0.0561 (10)	0.0129 (7)	0.0074 (7)	0.0042 (7)
C12	0.0585 (10)	0.0542 (9)	0.0621 (11)	0.0101 (7)	−0.0001 (8)	0.0062 (8)
C13	0.0482 (9)	0.0516 (9)	0.0629 (10)	0.0111 (7)	0.0015 (7)	0.0095 (7)
C14	0.0414 (8)	0.0522 (9)	0.0596 (10)	0.0134 (7)	0.0013 (7)	0.0090 (7)
C15	0.0518 (9)	0.0571 (9)	0.0629 (11)	0.0118 (7)	−0.0012 (8)	0.0091 (8)
C16	0.0642 (11)	0.0820 (13)	0.0599 (11)	0.0181 (9)	−0.0020 (8)	0.0110 (9)
C17	0.0624 (11)	0.0871 (13)	0.0783 (13)	0.0238 (10)	0.0067 (10)	0.0364 (11)
C18	0.0545 (10)	0.0579 (10)	0.0948 (14)	0.0149 (8)	0.0027 (9)	0.0262 (10)
C19	0.0514 (9)	0.0541 (9)	0.0727 (11)	0.0144 (7)	−0.0026 (8)	0.0101 (8)
C20	0.0449 (8)	0.0442 (8)	0.0612 (10)	0.0084 (6)	0.0009 (7)	0.0064 (7)
C21	0.0451 (8)	0.0546 (9)	0.0555 (10)	0.0136 (7)	0.0012 (7)	0.0053 (7)
C22	0.0555 (9)	0.0529 (9)	0.0624 (10)	0.0122 (7)	−0.0045 (8)	−0.0034 (8)
C23	0.0537 (9)	0.0454 (8)	0.0747 (12)	0.0110 (7)	−0.0006 (8)	0.0045 (8)
C24	0.0418 (8)	0.0465 (8)	0.0653 (10)	0.0139 (6)	0.0016 (7)	0.0095 (7)
C25	0.0592 (10)	0.0544 (9)	0.0807 (12)	0.0146 (8)	0.0026 (9)	0.0219 (9)
C26	0.0708 (12)	0.0732 (12)	0.0702 (12)	0.0192 (9)	0.0039 (9)	0.0255 (10)
C27	0.0689 (11)	0.0751 (12)	0.0568 (10)	0.0190 (9)	−0.0034 (8)	0.0085 (9)
C28	0.0585 (10)	0.0523 (9)	0.0604 (10)	0.0119 (7)	−0.0016 (8)	0.0050 (8)
C29	0.0418 (8)	0.0480 (8)	0.0563 (10)	0.0128 (6)	0.0008 (7)	0.0063 (7)
C30	0.0520 (9)	0.0614 (10)	0.0574 (10)	0.0104 (8)	0.0046 (7)	0.0045 (8)
C31	0.0550 (10)	0.0697 (11)	0.0607 (11)	0.0064 (8)	0.0034 (8)	0.0100 (8)
C32	0.0531 (9)	0.0526 (9)	0.0617 (11)	0.0094 (7)	0.0045 (8)	0.0084 (8)
C33	0.0511 (9)	0.0452 (8)	0.0593 (10)	0.0124 (7)	0.0021 (7)	0.0105 (7)
C34	0.0554 (9)	0.0561 (9)	0.0659 (11)	0.0124 (7)	0.0100 (8)	0.0159 (8)
C35	0.0761 (12)	0.0660 (11)	0.0601 (11)	0.0196 (9)	0.0090 (9)	0.0154 (8)
C36	0.0708 (12)	0.0614 (10)	0.0661 (11)	0.0182 (9)	−0.0082 (9)	0.0097 (8)
C37	0.0507 (9)	0.0572 (10)	0.0794 (12)	0.0122 (7)	−0.0023 (9)	0.0145 (9)
C38	0.0541 (9)	0.0588 (10)	0.0621 (10)	0.0131 (7)	0.0070 (8)	0.0168 (8)
O1	0.0676 (8)	0.0766 (8)	0.0653 (8)	−0.0072 (6)	0.0031 (6)	0.0079 (6)
O2	0.0733 (8)	0.0853 (9)	0.0667 (8)	−0.0125 (7)	−0.0006 (6)	0.0129 (6)

### Geometric parameters (Å, °)

**Table:**

C1—C2	1.370 (2)	C20—C21	1.374 (2)
C1—C10	1.412 (2)	C20—C29	1.412 (2)
C1—H1	0.9300	C20—H20	0.9300
C2—C3	1.417 (2)	C21—C22	1.419 (2)
C2—C11	1.493 (2)	C21—C30	1.490 (2)
C3—C4	1.360 (2)	C22—C23	1.358 (2)
C3—H3	0.9300	C22—H22	0.9300
C4—C5	1.404 (2)	C23—C24	1.407 (2)
C4—H4	0.9300	C23—H23	0.9300
C5—C6	1.415 (2)	C24—C25	1.415 (2)
C5—C10	1.420 (2)	C24—C29	1.424 (2)
C6—C7	1.356 (2)	C25—C26	1.356 (2)
C6—H6	0.9300	C25—H25	0.9300
C7—C8	1.397 (3)	C26—C27	1.400 (2)
C7—H7	0.9300	C26—H26	0.9300
C8—C9	1.356 (2)	C27—C28	1.362 (2)
C8—H8	0.9300	C27—H27	0.9300
C9—C10	1.414 (2)	C28—C29	1.415 (2)
C9—H9	0.9300	C28—H28	0.9300
C11—O1	1.2258 (18)	C30—O2	1.2184 (18)
C11—C12	1.471 (2)	C30—C31	1.485 (2)
C12—C13	1.325 (2)	C31—C32	1.324 (2)
C12—H12	0.9300	C31—H31	0.9300
C13—C14	1.460 (2)	C32—C33	1.460 (2)
C13—H13	0.9300	C32—H32	0.9300
C14—C15	1.391 (2)	C33—C34	1.392 (2)
C14—C19	1.397 (2)	C33—C38	1.399 (2)
C15—C16	1.383 (2)	C34—C35	1.374 (2)
C15—H15	0.9300	C34—H34	0.9300
C16—C17	1.375 (3)	C35—C36	1.375 (2)
C16—H16	0.9300	C35—H35	0.9300
C17—C18	1.376 (3)	C36—C37	1.374 (2)
C17—H17	0.9300	C36—H36	0.9300
C18—C19	1.377 (2)	C37—C38	1.371 (2)
C18—H18	0.9300	C37—H37	0.9300
C19—H19	0.9300	C38—H38	0.9300
			
C2—C1—C10	121.64 (14)	C21—C20—C29	121.38 (14)
C2—C1—H1	119.2	C21—C20—H20	119.3
C10—C1—H1	119.2	C29—C20—H20	119.3
C1—C2—C3	118.81 (14)	C20—C21—C22	119.00 (15)
C1—C2—C11	119.27 (14)	C20—C21—C30	119.14 (14)
C3—C2—C11	121.86 (14)	C22—C21—C30	121.86 (14)
C4—C3—C2	120.73 (15)	C23—C22—C21	120.81 (15)
C4—C3—H3	119.6	C23—C22—H22	119.6
C2—C3—H3	119.6	C21—C22—H22	119.6
C3—C4—C5	121.34 (15)	C22—C23—C24	121.21 (14)
C3—C4—H4	119.3	C22—C23—H23	119.4
C5—C4—H4	119.3	C24—C23—H23	119.4
C4—C5—C6	122.83 (15)	C23—C24—C25	122.72 (14)
C4—C5—C10	118.66 (14)	C23—C24—C29	118.78 (15)
C6—C5—C10	118.50 (15)	C25—C24—C29	118.49 (15)
C7—C6—C5	120.92 (16)	C26—C25—C24	121.06 (15)
C7—C6—H6	119.5	C26—C25—H25	119.5
C5—C6—H6	119.5	C24—C25—H25	119.5
C6—C7—C8	120.41 (17)	C25—C26—C27	120.53 (17)
C6—C7—H7	119.8	C25—C26—H26	119.7
C8—C7—H7	119.8	C27—C26—H26	119.7
C9—C8—C7	120.66 (17)	C28—C27—C26	120.38 (16)
C9—C8—H8	119.7	C28—C27—H27	119.8
C7—C8—H8	119.7	C26—C27—H27	119.8
C8—C9—C10	120.75 (16)	C27—C28—C29	120.85 (15)
C8—C9—H9	119.6	C27—C28—H28	119.6
C10—C9—H9	119.6	C29—C28—H28	119.6
C1—C10—C9	122.51 (14)	C20—C29—C28	122.54 (14)
C1—C10—C5	118.75 (14)	C20—C29—C24	118.78 (14)
C9—C10—C5	118.74 (15)	C28—C29—C24	118.68 (15)
O1—C11—C12	121.57 (15)	O2—C30—C31	121.21 (16)
O1—C11—C2	119.81 (15)	O2—C30—C21	120.69 (15)
C12—C11—C2	118.62 (13)	C31—C30—C21	118.09 (14)
C13—C12—C11	121.17 (15)	C32—C31—C30	121.54 (15)
C13—C12—H12	119.4	C32—C31—H31	119.2
C11—C12—H12	119.4	C30—C31—H31	119.2
C12—C13—C14	127.04 (15)	C31—C32—C33	127.23 (15)
C12—C13—H13	116.5	C31—C32—H32	116.4
C14—C13—H13	116.5	C33—C32—H32	116.4
C15—C14—C19	118.01 (16)	C34—C33—C38	118.17 (15)
C15—C14—C13	119.50 (14)	C34—C33—C32	119.13 (14)
C19—C14—C13	122.48 (15)	C38—C33—C32	122.70 (15)
C16—C15—C14	120.84 (16)	C35—C34—C33	120.51 (16)
C16—C15—H15	119.6	C35—C34—H34	119.7
C14—C15—H15	119.6	C33—C34—H34	119.7
C17—C16—C15	120.22 (17)	C34—C35—C36	120.51 (17)
C17—C16—H16	119.9	C34—C35—H35	119.7
C15—C16—H16	119.9	C36—C35—H35	119.7
C16—C17—C18	119.73 (18)	C37—C36—C35	119.78 (17)
C16—C17—H17	120.1	C37—C36—H36	120.1
C18—C17—H17	120.1	C35—C36—H36	120.1
C17—C18—C19	120.50 (16)	C38—C37—C36	120.42 (16)
C17—C18—H18	119.8	C38—C37—H37	119.8
C19—C18—H18	119.8	C36—C37—H37	119.8
C18—C19—C14	120.70 (16)	C37—C38—C33	120.61 (16)
C18—C19—H19	119.7	C37—C38—H38	119.7
C14—C19—H19	119.7	C33—C38—H38	119.7
			
C10—C1—C2—C3	−0.5 (2)	C29—C20—C21—C22	−0.4 (2)
C10—C1—C2—C11	−177.88 (13)	C29—C20—C21—C30	−179.62 (13)
C1—C2—C3—C4	−1.7 (2)	C20—C21—C22—C23	−1.7 (2)
C11—C2—C3—C4	175.60 (14)	C30—C21—C22—C23	177.56 (14)
C2—C3—C4—C5	2.1 (2)	C21—C22—C23—C24	2.1 (3)
C3—C4—C5—C6	179.33 (14)	C22—C23—C24—C25	178.04 (15)
C3—C4—C5—C10	−0.3 (2)	C22—C23—C24—C29	−0.5 (2)
C4—C5—C6—C7	−179.29 (15)	C23—C24—C25—C26	−177.69 (15)
C10—C5—C6—C7	0.3 (2)	C29—C24—C25—C26	0.9 (2)
C5—C6—C7—C8	0.4 (3)	C24—C25—C26—C27	−0.2 (3)
C6—C7—C8—C9	−0.3 (3)	C25—C26—C27—C28	−0.7 (3)
C7—C8—C9—C10	−0.6 (3)	C26—C27—C28—C29	0.9 (3)
C2—C1—C10—C9	−178.13 (14)	C21—C20—C29—C28	−177.50 (14)
C2—C1—C10—C5	2.3 (2)	C21—C20—C29—C24	1.9 (2)
C8—C9—C10—C1	−178.34 (15)	C27—C28—C29—C20	179.19 (14)
C8—C9—C10—C5	1.2 (2)	C27—C28—C29—C24	−0.2 (2)
C4—C5—C10—C1	−1.9 (2)	C23—C24—C29—C20	−1.5 (2)
C6—C5—C10—C1	178.51 (13)	C25—C24—C29—C20	179.91 (13)
C4—C5—C10—C9	178.52 (14)	C23—C24—C29—C28	177.96 (14)
C6—C5—C10—C9	−1.1 (2)	C25—C24—C29—C28	−0.7 (2)
C1—C2—C11—O1	26.3 (2)	C20—C21—C30—O2	27.6 (2)
C3—C2—C11—O1	−150.94 (16)	C22—C21—C30—O2	−151.59 (17)
C1—C2—C11—C12	−153.52 (15)	C20—C21—C30—C31	−152.83 (15)
C3—C2—C11—C12	29.2 (2)	C22—C21—C30—C31	27.9 (2)
O1—C11—C12—C13	14.7 (3)	O2—C30—C31—C32	12.3 (3)
C2—C11—C12—C13	−165.43 (15)	C21—C30—C31—C32	−167.26 (15)
C11—C12—C13—C14	−178.11 (14)	C30—C31—C32—C33	−177.63 (15)
C12—C13—C14—C15	−166.36 (16)	C31—C32—C33—C34	−168.95 (16)
C12—C13—C14—C19	12.8 (3)	C31—C32—C33—C38	11.4 (3)
C19—C14—C15—C16	−0.8 (2)	C38—C33—C34—C35	−0.7 (2)
C13—C14—C15—C16	178.42 (14)	C32—C33—C34—C35	179.67 (15)
C14—C15—C16—C17	0.7 (3)	C33—C34—C35—C36	0.2 (3)
C15—C16—C17—C18	−0.1 (3)	C34—C35—C36—C37	0.3 (3)
C16—C17—C18—C19	−0.4 (3)	C35—C36—C37—C38	−0.3 (3)
C17—C18—C19—C14	0.3 (3)	C36—C37—C38—C33	−0.2 (3)
C15—C14—C19—C18	0.3 (2)	C34—C33—C38—C37	0.7 (2)
C13—C14—C19—C18	−178.88 (14)	C32—C33—C38—C37	−179.68 (14)

### Hydrogen-bond geometry (Å, °)

**Table:**

*D*—H···*A*	*D*—H	H···*A*	*D*···*A*	*D*—H···*A*
C13—H13···O1	0.93	2.49	2.814 (2)	101
C32—H32···O2	0.93	2.50	2.820 (2)	101
C18—H18···Cg1^i^	0.93	2.98	3.644	130
C15—H15···Cg2^ii^	0.93	2.94	3.642	134
C37—H37···Cg3^iii^	0.93	2.96	3.610	128
C1—H1···Cg5^ii^	0.93	2.97	3.611	127
C6—H6···Cg6^iii^	0.93	2.92	3.583	130

Symmetry codes: (i) −*x*+1, −*y*, −*z*+1; (ii) −*x*+1, −*y*+1, −*z*+1; (iii) −*x*, −*y*+1, −*z*+1.
